# Precision farming in aquaculture: non-invasive monitoring of Atlantic salmon (*Salmo salar*) behaviour in response to environmental conditions in commercial sea cages for health and welfare assessment

**DOI:** 10.3389/frobt.2025.1574161

**Published:** 2025-04-23

**Authors:** Meredith Burke, Dragana Nikolic, Pieter Fabry, Hemang Rishi, Trevor Telfer, Sonia Rey Planellas

**Affiliations:** ^1^ Institute of Aquaculture, University of Stirling, Stirling, United Kingdom; ^2^ Observe Technologies, Richmond, United Kingdom

**Keywords:** Atlantic salmon, aquaculture, temperature, photoperiod, storm event, fish distribution, machine learning

## Abstract

Studies show that Atlantic salmon in captivity adjust their distribution in sea cages based on environmental gradients like temperature, waves, and photoperiod. This study used a computer vision algorithm at three marine farms to analyse fish group swimming behaviour termed “activity” (measured in percent), which includes fish abundance, speed, and shoal cohesion. The activity metric inferred the depth distribution of the main fish group and was analysed with respect to environmental conditions to explore potential behavioural drivers and used to assess changes in fish behaviour in response to a stressor, a storm event. During winter conditions, Farms A and B showed distinct thermal stratification, with fish activity demonstrating preference for the warmer lower water column (39.6 ± 15.3% and 27.5 ± 10.2%) over the upper water column (16.3 ± 5.7% and 18 ± 3.3%; p < 0.001). At Farm C, with thermally homogenous water, fish activity was similarly distributed between the upper (18.2 ± 6.9%) and lower (17.7 ± 7.6%) water column. Severe weather increased wave heights, influencing fish horizontal distribution differently at Farms B and C. At Farm B, a deeper site, fish remained in the warmer lower water column and avoided surface waves, while at Farm C, with shallower cages, they moved toward the side of the cage nearest the centre of the farm, presumably less exposed due to nearby cages. Understanding fish behavioural responses to environmental conditions can inform management practices, while using cameras with associated algorithms offers a powerful, non-invasive tool for continuously monitoring and safeguarding fish health and welfare.

## 1 Introduction

The study of animal behaviour has been of interest since the time of Aristotle, and has long been used in agricultural settings to enhance animal welfare (Fraser and Broom, 1997). However, in recent decades, technological advancements have greatly improved the aquaculture industry’s capacity to study and monitor aquatic species (Colgan and Salmon, 1986; Feng et al., 2024). The inherent challenges associated with monitoring underwater animals has pushed the development of innovative underwater technologies. Most commonly, fish tagging and acoustic telemetry have become essential tools for tracking fish movements and distribution to monitor ecosystem health and for conservation purposes (Lopez-Marcano et al., 2021).

In sea cage aquaculture systems, the challenges of poor visibility (e.g., from water turbidity, fish density, variable lighting conditions), and restricted access, due to the remote location of many farms, further emphasise the need for arrays of sophisticated technologies to monitor fish effectively under farming conditions (Føre et al., 2018). There are many technologies that can be employed for farmers to monitor their fish such as biosensors, fish telemetry, hydroacoustic sensors (echosounders) and cameras, with the latter two being the most prominent in commercial sea cages (Føre et al., 2018; O’Donncha et al., 2021; Georgopoulou et al., 2021). Atlantic salmon (*Salmo salar*) producers are at the forefront of technological advancements in this domain. It has become increasingly important to monitor the behaviour of salmon in production cages, as it plays a crucial role in ensuring good welfare practices (Føre et al., 2017). For example, fish exhibit behavioural responses to various environmental stimuli, such as light, water quality, stocking density, and current flow. These responses can manifest as changes in swimming activity, feeding patterns, or aggressive interactions with conspecifics, providing farmers with valuable insights into fish welfare and stress levels (An et al., 2021; Barreto et al., 2022). Monitoring fish distribution throughout the cage, as a behavioural parameter, in relation to various environmental and health-related parameters also allows for more effective and informed management practices, ultimately contributing to the sustainability and success of aquaculture operations. The distribution of Atlantic salmon within production cages is influenced by a variety of factors, with temperature playing a pivotal role. Fish are ectotherms and display a high sensitivity to even slight temperature variations (Pankhurst and Munday, 2011). Adult Atlantic salmon prefer temperatures between 13°C–18°C and position themselves in the warmest available waters when temperatures are below 14°C (Oppedal et al., 2011; Korus et al., 2024). Ectotherms, like fish, use behavioural thermoregulation, to seek out preferred thermal environments as they are mostly unable to regulate their body temperature internally (Johansson et al., 2009).

Other forms of behavioural thermoregulation are emotional and behavioural fever in fish, wherein fish may respond to stress or illness by physically seeking warmer temperatures (Boltaña et al., 2013; Rey et al., 2015; Huntingford et al., 2020), similar to the endotherms physiological or emotional fever (e.g., stress induced hyperthermia; Olivier et al., 2003; Oka et al., 2018). Additionally, there is evidence of fish showing thermal preferences associated with their circadian rhythm, with zebrafish and Nile tilapia preferring higher temperatures in the second half of the light phase and lower temperatures at the end of the dark phase (Vera et al., 2023). Similarly, studies have found that in general, salmon show a diurnal depth preference with a tendency to swim deeper during the day to avoid high light intensity (negative phototaxis), while at night they swim closer to the surface (Huse and Holm, 1993; Oppedal et al., 2001; Oppedal et al., 2007; Johannesen et al., 2022).

At the individual level, differences in stress coping style also influence salmon behaviour in sea cages and will likely impact their position in the cage. Fish exhibit two stress coping styles: proactive and reactive. Proactive fish will take risks, explore and have high feed motivation. In contrast, reactive fish are shyer and tend to exhibit behaviours opposite to their proactive counterparts (Castanheira et al., 2017). There are also intermediate fish, often the most abundant group within the populations, that are not as consistent and may change preferences depending on the external and internal conditions (Ferrari et al., 2020). In the context of fish distribution, proactive fish have been observed to actively avoid hypoxic regions compared to reactive fish which tend to persist in these areas rather than escape from it to move to a novel, better oxygenated environment (Damsgård et al., 2019). Proactive animals also tend to be more dominant and prefer higher temperatures probably due to higher metabolic rates (Cerqueira et al., 2016).

Other oceanographic processes may impact fish distribution in sea cages, such as current speed, direction and wave height. Salmon in cages tend to swim in a circular pattern around the cage, following the net shape (Juell and Westerberg, 1993; Oppedal et al., 2011) or show positive rheotaxis swimming against the current and holding position (Webb and Cotel, 2011). For example, Johannesen et al. (2022) observed that salmon in their study exhibited positive rheotaxis, moving toward the region of the cage most exposed to currents, possibly as a form of environmental enrichment that simulates natural swimming behaviours and helps reduce stress. Cage deformation plays a role in fish distribution as well, as strong water velocities can change the shape of the nets reducing cage volume and thus reduces space for salmon to occupy (DeCew et al., 2013). Recently, the effects of waves on salmon behaviour and welfare in sea cages has gained attention with the growing interest in offshore aquaculture and more dynamic environments (Hvas et al., 2021; Morro et al., 2022; Szewczyk et al., 2024). Studies have shown that while fish tend to prefer areas of the cage exposed to higher currents, they typically avoid the near-surface when waves are high (Johannesen et al., 2020; Johannesen et al., 2022; Klebert et al., 2023). However, exposing fish to turbulence similar to the effects of waves under laboratory conditions show no major effects on the final welfare or growth of salmon (Barbier et al., 2024; Athammer et al., 2024). It is also important to recognise the significance of the farm orientation, as cage blocks are typically aligned with the longer side in the streamwise direction. This can lead to significant variations in water flow, with some cages experiencing highly variable or more consistent flow patterns depending on their location within the cage block relative to the tidal cycle (Klebert et al., 2013; Burke et al., 2021). Feeding regime also influences fish distribution, as fish tend to ascend to the surface and centre of the cage with increased appetite and while feeding and subsequently descend and move towards the periphery of the cage when satiated (Juell et al., 1994; Oppedal et al., 2011).

Stress-related behavioural responses are also evident in aquatic species. Notably, fish tend to congregate in response to predator or environment-related stressors. In these cases, the fish will shoal, forming a tightly grouped and cohesive unit, as a stress-defence mechanism (Järvi and Uglem, 1993; Hoare et al., 2004). Understanding the factors driving shoaling in cultured fish species is important, as this behaviour can create hypoxic conditions in areas where fish cluster within the cage. Moreover, close proximity among fish can lead to physical injury and may also facilitate the transmission of diseases or parasites among conspecifics (Bui et al., 2019).

Precision fish farming, a concept defined as the use of technology and automation to improve decision making in aquaculture, has come to the forefront of the industry (Føre et al., 2018). This is improving farmers’ capabilities to observe, monitor and increase control over their fish and surrounding environment. More sophisticated and reliable sensors are available for aquaculture purposes that can measure water quality parameters like temperature, oxygen, nitrogen, salinity and turbidity. With the advent of computer vision techniques, there has been an increase in studies using cameras and imaging-base systems with algorithms to extract and analyse the data procured as a non-invasive, cost-effective means for tracking and monitoring fish in aquaculture (Zion, 2012; Qian et al., 2016; An et al., 2021; Barreto et al., 2022; Georgopoulou et al., 2021; Vo et al., 2021).

In the present study, underwater cameras were deployed, and the real-time videos were analysed using a machine-learning algorithm that converts the videos into a numerical format as a proxy for fish abundance and shoal cohesion. The aim of this study was to understand fish distribution patterns related to the environmental conditions within the sea cages. The hypothesis was that fish would follow vertical thermal gradients and change horizontal distribution based on hydrodynamics. Gaining a more profound insight into the distribution patterns of fish under typical conditions provides farmers with a valuable baseline to then discern whether deviations in these behaviours can be attributed to stressors of environmental (e.g., storm events or hypoxic conditions) or health origins (e.g., sea lice or gill health issues; Sadoul et al., 2014).

## 2 Materials and methods

### 2.1 Study sites

**FIGURE 1:**
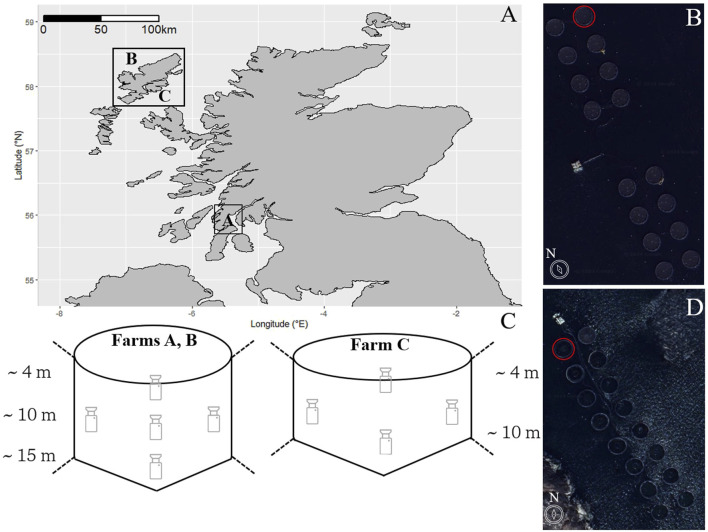
**(A)** Study sites: A map of Scotland showing the locations of the Atlantic salmon aquaculture farms in western Scotland. Satellite images (from Google Earth) depicting the layouts of Farms A/B **(B)** and Farm C **(D)**, where red circles indicate the study cages. **(C)** Schematic showing the layout of the cameras within the study cages.

Three commercial Atlantic salmon farms in Scotland were used for this study, one was located within a small bay in a sea loch on the west coast of Scotland (Farm A), while the other two were located on the outer (Farm B) and inner (Farm C) coasts of the Western Isles (Figure 1A). All farms consist of 16 circular cages arranged in two parallel rows. Farms A and B contained cages with a circumference of 90 m and a cylindrical depth of 15 m (mesh size: 18 mm, volume: 11, 915 m^3^), transitioning into a conical bottom that extends to a total depth of 18 m, and are each equipped with predator nets. Farm C consisted of cages with a circumference of 120 m and a cylindrical depth of 10 m (mesh size: 18 mm, volume: 11, 460 m^3^), tapering into a conical bottom that extends to a total depth of 12 m. The farms contained adult post-smolt salmon with an average weight of 3.7 kg, 2.1 kg, and 1.8 kg for Farm A, B, and C, respectively. The fish were sourced from two different broodstocks, Farms A and B were from the same stock, which differed from Farm C. All fish had been transferred into their respective farms in October 2021, February 2023 and May 2023, as ∼100 g smolts from the two hatcheries.

Farm A has a seabed depth of 25–30 m (I-Boating: Marine Charts and Gps, 2024) and dominant semi-diurnal tides with a 2 m range. The water temperatures range from an average of 14.4°C ± 0.4°C in the summer to 7.2°C ± 0.5°C in winter (average values for the period 2014–2023 from seatemperature.info). Farm B has a seabed depth of 23–27 m, with semi-diurnal tides of 2.5 m range. The temperatures range from 13.8°C ± 0.5°C in the summer and 7.6°C ± 0.5°C in the winter. Farm C is the shallowest of the three sites, with a depth of only 15–20 m, and semi-diurnal tides of 3 m range. The temperatures at this site range from 14.7°C ± 0.4°C in the summer and 7.2°C ± 0.5°C in the winter. Dominant currents relevant to the cage depth occur in parallel to the orientation of all farms.

### 2.2 Data collection

There were 5 and 4 video camera modules (Sony model: IPCM-3516DS385-D29-AZ3611) installed by AKVA group (www.akvagroup.com) in a single, randomly allocated cage for farms A/B and C, respectively. The five camera systems were positioned facing upwards with three down the centre (approximately 4 m, 9 m, 14 m), and 2 at 9 m on the inner and outer areas of the cage, respectively, while the 4-camera system did not have the 14 m camera (Figure 1B). While the cameras were deployed at predetermined depths, their actual depths varied due to routine removal for cleaning procedures. Each camera was equipped with a pressure sensor to verify its depth upon redeployment and ensure consistent spacing between cameras. This pressure data was also used to determine fish depth preference.

The camera system used in this study was optimised to focus on objects within a range of 20–30 cm up to 200 cm. Beyond this distance, the image extends to infinity but without maintaining sharp focus. This focal range was selected to balance close-range behavioural observations with broader spatial coverage. Therefore, the cameras positioned 5 m apart along the centre were unlikely to have overlapping fields of view, given the limited underwater visibility and fish density typical of aquaculture systems. The cameras recorded during daylight (8:30–16:30 in January, 7:00–19:00 by May) at 25 frames per second, with a resolution of 1920 × 1,080 pixels and a video bit rate of 16 Mb per second. The photoperiod was accessed from Time and Date AS (timeanddate.com). Continuous temperature was recorded by RDO PRO-X sensors (accuracy of ±0.1°C and resolution of 0.01°C) attached to each camera.

Innovex (https://www.innovex.cl/) current sensors and weather stations were deployed at Farms B and C between January and May 2024, collecting data every 5 min to capture environmental conditions, including a storm event (Storm Isha: January 21–22, 2024). Farm A was excluded from this deployment as the fish had been harvested prior to this period. The current sensor was placed at 5 m depth off the barge to gather current speeds and directions, while the weather station collected wind speed and direction, wave height, and air pressure. As an additional fish performance and welfare indicator, daily Specific Feeding Rate (SFR) was extrapolated by farm staff daily as the % body weight fed:

$$\frac{\text{feed}\;\text{given}}{\text{fish}\;\text{biomass}}\times 100$$

where biomass was determined through sample and mortality weights and number of fish in each cage.

Health-related parameters were not factored into this study as it was undertaken during the winter months, a period when gill health issues and other parasitic burdens are relatively low. In this case, there was no disease outbreak, as confirmed by farm staff. Treatments can also affect fish behaviour, as they are a source of stress (Lieke et al., 2020). Distribution data on the rare occasions treatments were administered were excluded from the analysis.

### 2.3 Data analysis

**FIGURE A1:**
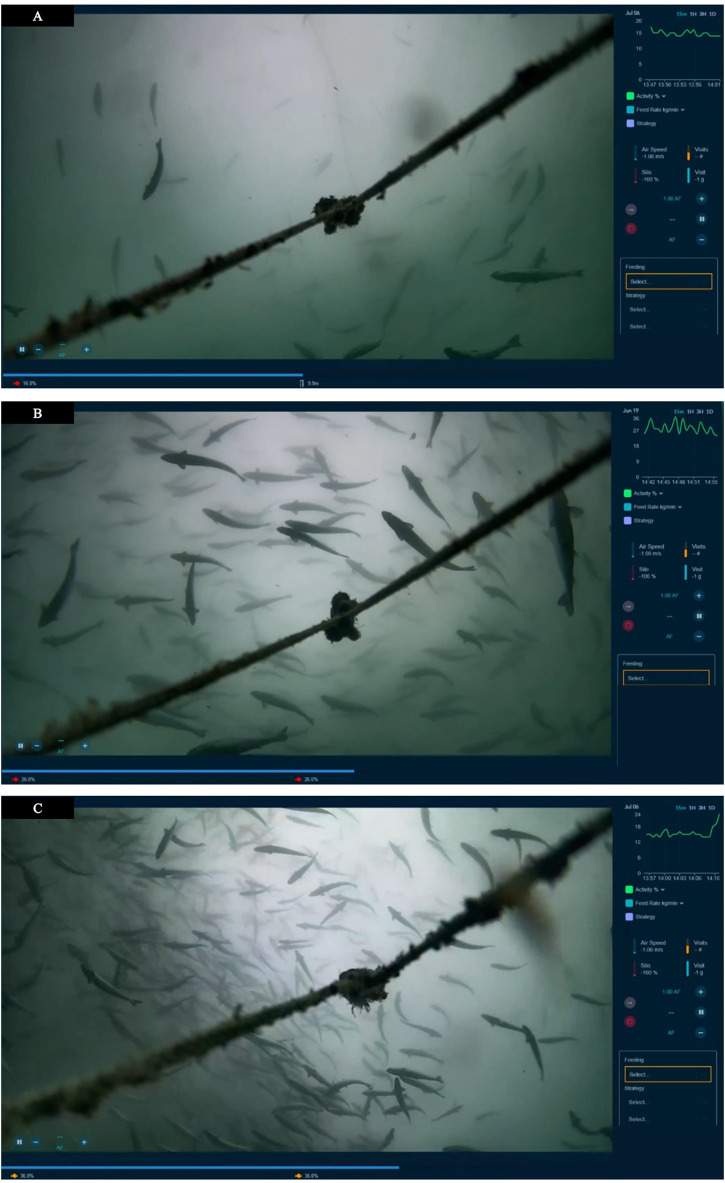
Screenshots of three different camera views, with associated AI-generated activity level (red fish in bottom left of each panel, and a time series in green on the top right of each panel). Each panel increases in activity from **(A)** (16%), **(B)** (26%) and **(C)** (36%).

The video recordings were analysed via an algorithm developed by Observe Technologies (www.observe.tech). The algorithm processes the video footage and outputs behavioural data useful for farmers. Observe Technologies uses a patented (EP3644717) convolutional neural network for the heuristic estimation of fish activity. The approach leverages various discrete features characterising fish behaviour, including but not limited to, cohesive schooling, average distance from camera, number of fish on screen, and fish speed. The model is trained globally rather than tracking individual fish, learning patterns from labelled data. Since all features share the same network architecture up to the final layer, their individual contributions to the final activity score are not explicitly defined. The model iteratively adjusts its weights using standard deep-learning optimisation techniques, producing a normalised relative activity score (0%–100%). Comprehensive model evaluations are performed using training, validation and test sets, in line with best practices in deep learning. Additionally, domain experts in aquaculture continuously validate the model’s outputs to ensure real-world applicability (Table A1). Separately, for this study, the real-time output was also examined by experts to verify its accuracy before it was provided for analysis. An example of the algorithm's output, illustrated as screenshots from analysed videos, is provided in the Appendix (Figure A1). The algorithm was independently validated by two observers from the University of Stirling, using videos recorded using the same AKVA feed cameras. For this study, the algorithm’s abundance estimation component was specifically validated. Fish abundance in video frames was quantified and correlated with the algorithm’s ‘activity’ output using Pearson correlation (α = 0.05) across 55 randomly selected video stills. Interobserver reliability was assessed using the Intraclass Correlation Coefficient (ICC). While the full algorithm incorporates additional behavioural features (e.g., shoaling patterns and swimming speed), abundance is the key component relevant to this study, as it determines depth and location preferences.

Statistical analyses were conducted to examine the variations in activity throughout the 5 months sampling period, spanning from winter to spring in 2023 and 2024 (from 13th January to 1st May 2023 [Farm A] and 1st January – 1st May 2024 [Farms B and C]). The algorithm derived data (activity, *a*) was originally sampled at 5 s then averaged hourly to reduce excess noise. Additionally, fish activity during feeding was excluded from the analysis to prevent potential bias. At each time point, the camera detecting the highest activity level was identified, and its corresponding depth, as measured by pressure sensors, was recorded as the depth of maximum activity. This depth was used to infer the preferred depth of the majority of the fish population.

Non-parametric Kolmogorov-Smirnov (K-S) tests were used to assess significant differences in temperature between the upper and lower water column, based on temperature data recorded by sensors attached to the cameras. Similarly, variations in fish activity across depths were analysed using differences in activity recorded by the vertically positioned cameras, with depth determined by their integrated pressure sensors. Additionally, an Ordinary Least Squares (OLS) regression was utilised to examine the relationship between photoperiod and fish depth. For the oceanographic conditions, K-S tests were also employed to test the significance in wind, current, and wave heights around storm Isha, and how these affected the fish distribution (fish activity levels) horizontally across the cages at Farms B and C. All statistics were conducted in Python programming language. Raw data is available through DataSTORRE (http://hdl.handle.net/11667/242).

## 3 Results

### 3.1 Validation of the activity algorithm

Results of the algorithm validation show that there was a strong correlation (R^2^ = 0.70, p < 0.001) between the abundance of fish counted in the feeding cameras by human observers to the still images from the same videos used to calculate activity by the algorithm. The ICC was calculated to determine both the absolute agreement (ICC = 0.99) and the consistency (ICC = 0.98) between the two observers. Both reflect strong agreement between the observers that was statistically significant (p < 0.001). While the other specific components of the algorithm could not be separately validated, it is assumed that the remaining 30% of unexplained variation is associated with these components.

### 3.2 Vertical fish distribution in stratified waters

**FIGURE 2:**
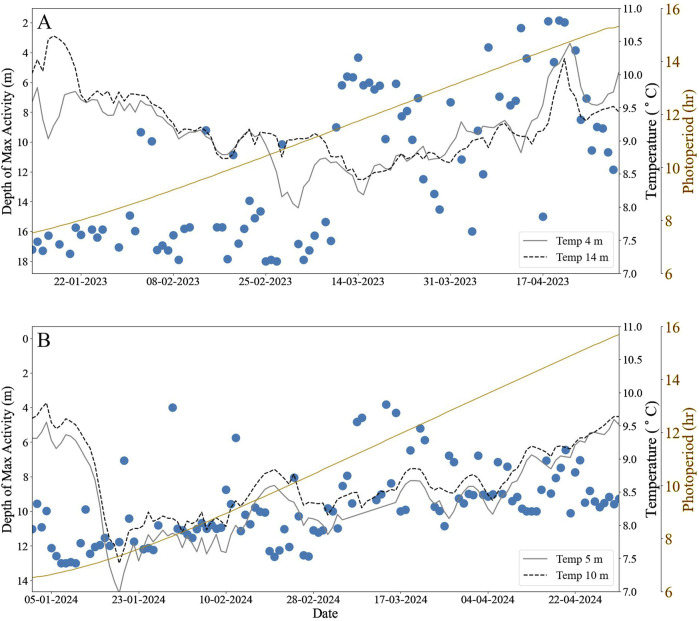
The daily average depth (m) of maximum salmon activity during daylight hours (blue) with temperature at 2 depths at Farm A [**(A)**; grey: 4 m; black dotted: 14 m] and Farm B [**(B)**; grey: 5 m; black dotted: 10 m]. Yellow lines indicate the daily photoperiod (hours) on the secondary y-axis for each site.

**FIGURE 3:**
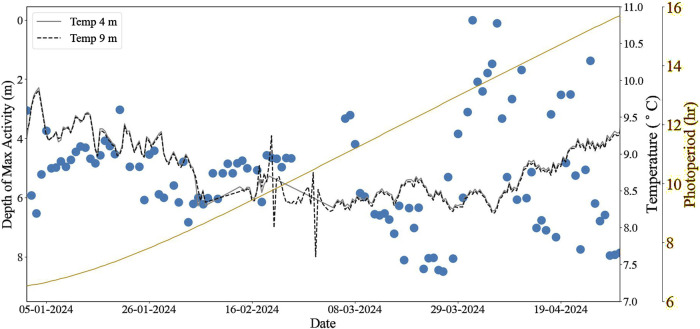
The daily average depth (m) of maximum salmon activity during daylight hours (blue) at Farm C, with temperature at 2 depths overlaid (grey: 4 m; black dotted: 9 m) and daily photoperiod (hours) shown in yellow on the secondary y-axis.

The vertical distribution of fish during daylight hours was explored through the differences in fish activity levels observed by cameras at different depths, primarily through the cameras placed at approximately 14 m depth (heinafter referred to as “lower water column (LWC)” of the cage) and the camera at 4 m depth (“upper water column (UWC)”). At Farm A, there was a notable shift in the fish distribution between January and May 2023. Initially, there was significantly higher activity in the LWC (*a* = 39.6% ± 15.3%) in contrast to the UWC (*a* = 16.3% ± 5.65%; D = 0.99, p < 0.001; Figure 2A). The mean fish depth of maximum activity was 14.9 ± 4.7 m until March 10^th^ 2023, at which point there was a significant decrease in the mean depth of maximum fish activity to 7.5 ± 5.3 m (D = 0.78, p < 0.001; Figure 3A). This shift coincided with changes in seawater temperature. Before March 10th, the LWC maintained a higher temperature compared to the UWC (9.4°C ± 0.45°C and 9.1°C ± 0.49°C, respectively). However, post-March 10th, the UWC warmed notably to an average of 9.2°C ± 0.6°C (with a maximum of 10.4°C), surpassing the average temperature of the LWC (9.0°C ± 0.1°C). Consequently, the fish adjusted their distribution, favouring the UWC and mid-depth zones for the remainder of the study period. Additionally, while the maximum activity was located in the LWC, there was an intermediate level of activity (*a* = 21.5 ± 10.2%) in the centre of the cage, and this remained for the entirety of the study period.

Photoperiod had an accompanying effect on fish depth preference, as an OLS regression analysis revealed a significant relationship between photoperiod and fish depth (β = −1.4, R^2^ = 0.47, p < 0.001). As the length of day increases to equal or greater than length of night (>12 h) the fish tended towards shallower depths.

At Farm B, waters were stratified for the duration of the study period, with LWC warmer than UWC. At the beginning of the study period in January 2024, the UWC temperature was 9.3°C ± 0.1°C while the LWC temperature was 9.6°C ± 0.1°C. These then decreased on January 19th and over the following 10 days to 6.9°C and 7.4°C for UWC and LWC, respectively. Subsequently, there was a consistent increase in both temperatures up to 9.2°C ± 0.2°C and 9.3°C ± 0.2°C for UWC and LWC waters respectively, by the end of the study period, narrowing the difference between the two water layers. The mean fish activity was significantly higher at the bottom-most camera (*a* = 27.5 ± 10.2%) compared to that nearest the surface (*a* = 18 ± 3.3%; D = 0.52, p < 0.001), with the depth of maximum activity recorded at 11.0 ± 1.7 m. Additionally to note, as in Farm A, while the maximum activity was located in the LWC, an intermediate level of activity (*a* = 21.5 ± 9.1%) was observed on the inner side of the cage. This pattern remained stable until March 6th, 2024, when the average activity levels at both 4 m and 14 m converged (*a* = 23.5 ± 7.7% and *a* = 21.3 ± 4.3%, respectively; D = 0.24, p = 0.08). Concurrently, the depth of maximum activity decreased to 8.4 ± 1.7 m, and while it is significant (D = 0.75, p < 0.001), the fish remained deeper than in Farm A.

The change in photoperiod between the beginning and end of the study was higher in Farm B (9 h 10 m) compared to Farm A (7 h 47 m). While there was a statistically significant correlation between photoperiod and fish depth, the OLS regression revealed an R^2^ lower than Farm A at 0.27 (β = −4.0, p < 0.001).

### 3.3 Vertical fish distribution in mixed waters

At Farm C, the waters throughout the cage were unstratified throughout the entire study period, exhibiting uniform temperature in the vertical and horizontal dimensions of the cage, beginning with 9.3°C January 1st, 2024 (Figure 3). The waters then cooled to 8.5°C and 8.6°C for UWC and LWC, respectively, by February 20th 2024, and subsequently the whole water column increased to 9.1°C ± 0.2 °C by the end of the study period. The average depth of maximum activity was 5.0 ± 0.8 m until February 23rd, 2024, and subsequently there was a significant increase of maximum depth to 5.4 ± 2.3 m for the remainder of the study period (D = 0.36, p < 0.001), though the fish were well spread throughout the water column as indicated by the increased standard deviation. Fish activity was similar between the UPW and LWC throughout the study period (*a* = 18.2 ± 6.9% in UWC and *a* = 17.7 ± 7.6% in LWC) prior to February 23rd, increasing slightly (*a* = 20.1 ± 11.2% and *a* = 20.6 ± 9.4%, respectively) for the remainder of the study period.

As the photoperiod lengthened, the fish increasingly utilised more of the water column, with this effect becoming more pronounced when daylight duration equalled or exceeded nighttime. The increased standard deviation of fish depth (0.8 m before compared to 2.3 m after February 23rd) indicates greater cohesion during darker periods, whereas as the photoperiod lengthened, fish dispersed more evenly throughout the cage, utilising a larger portion of the available space. In contrast to Farms A and B, there is no statistical correlation between fish depth and photoperiod, as the fish both descend and ascend with more daylight hours ((β = −0.5, R^2^ = 0.01, p = 0.3).

### 3.4 Storm effects on horizontal fish distribution

#### 3.4.1 Fish response to conditions at farm B

**FIGURE 4:**
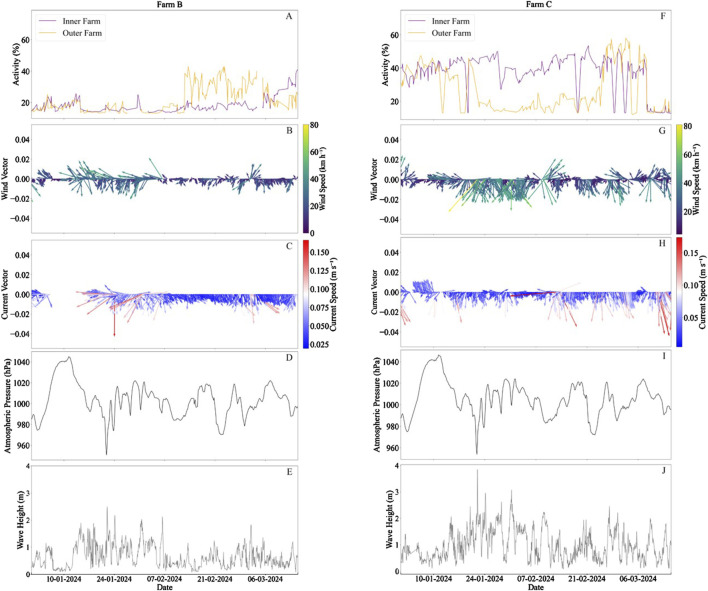
Fish behaviour and associated oceanographic conditions at Farms B (left) and C (right) including: 3-h averaged fish activity (%); **(A, F)**, wind speed (km h^−1^) and direction **(B, G)**, current speed (m s^−1^) and direction **(C, H)**, atmospheric pressure [hPa; **(D, I)**] and wave height [m; **(E, J)**]. Arrows indicate direction, with north at 0°.

Fish activity differed between the two sides of the cage under observation. At the start of the study there was little activity on either side of the cage (*a* = 16.3 ± 3.8% and *a* = 16.5 ± 3.0% for outer and inner regions of the cage, respectively; Figure 4A). At this point, the fish were primarily located it the LWC and centre regions of the cage, with average activities of 31.5% ± 10.9% and 25.8% ± 9.6%, respectively. The fish remained in these locations throughout a storm event (Storm Isha) which was indicated by a drop in air pressure from a maximum of 1,046.4 hPa to a minimum of 953.3 hPa (Figures 4D, I). The wind exhibited relatively high speeds at the beginning of the study (16.6 ± 11.4 km h^−1^) increasing to 24.7 ± 10.9 km h^−1^ in the south-southwest direction during the storm event (January 22–23; Figure 4B). The current speeds were also relatively high due to the storm event at 0.07 ± 0.03 m s^−1^ in the south-southeast direction (Figure 4C). Finally, the wave height was relatively high in the period during and after the storm (between January 22 and February 3) at 0.1 ± 0.4 m (Figure 4E). SFR was monitored throughout this time period, and it was similar the month prior to and during the storm at 0.81 ± 0.3 and 0.80 ± 0.01, respectively.

#### 3.4.2 Fish response to conditions at farm C

In Farm C, at the start of the study period, fish activity was high on both sides of the cage (*a* = 38.0 ± 7.3% and *a* = 34.2 ± 9.8%, for inner and outer farm regions respectively; Figure 4F). There was then a movement of fish to the region of the cage closest to the inner farm on January 22, when Storm Isha occurred, indicated by a significant increase in fish cohesion and activity in the inner area (*a* = 41.1 ± 7.2%; D = 0.13, p < 0.001) and a significant decrease in activity in the outer area (*a* = 17.9 ± 5.7%; D = 0.66, p < 0.001). This co-occurred with a significant increase in wind speeds from 19.4 ± 10.9 km h^−1^ in the southwest direction to 26.8 ± 15.3 km h^−1^ in the south direction (D = 0.27, p < 0.001; Figure 4G). Moreover, the fish at Farm C experienced a drop in their SFR from 0.66 to 0.29 during the storm, which is below the average from the 3 weeks prior (0.55 ± 0.13). This change to feeding rate was not observed in Farm B. Following February 25, the activity on both sides of the cage became similar for the remainder of the study period.

## 4 Discussion

### 4.1 Vertical distribution

Fish, like other ectothermic animals, possess a unique physiological characteristic – their internal body temperatures closely mirror the temperatures of the water they inhabit. Unlike endothermic animals (such as mammals and birds) that generate and regulate their own body heat, most fish rely on the ambient water temperature to govern their internal temperature (Abram et al., 2017; Clarke, 2017). This dependency on external thermal conditions evolved a behavioural adaptation known as behavioural thermoregulation (Mortensen et al., 2007; Abram et al., 2017; Clarke, 2017). This is the active and directed movement of fish to specific water temperatures that align with their internal physiological demands. During winter, the loch and bay of Farms A and B exhibit typical winter thermal stratified conditions with cooler waters above the thermocline and warmer, saltier waters below (Johnsen et al., 2020). As the air temperatures increase, the upper water column warms and this results in convection, mixing of the water column and de-stratifying the waters during the spring (Cannon et al., 2019; Cherif et al., 2023). Farm C however, located in a large shallow bay, was well mixed and remained unstratified throughout the study period. The present study shows that there was higher activity and thus density of fish in the LWC compared to the UWC during winter conditions at Farms A and B. At Farm C, there was no difference in temperature throughout the cage, resulting in the fish preferring the central region of the cage. Though the temperature gradients throughout the cages were minimal, fish can detect very slight changes in temperature, as low as 0.03°C (Beitinger and Fitzpatrick, 1979; Bartolini et al., 2015). Previous studies have shown that Atlantic salmon prefer temperatures between 14°C–18°C (Oppedal et al., 2011; Gamperl et al., 2021; Korus et al., 2024). Therefore, in the presence of temperature gradients within the cage, they are likely to preferentially occupy the warmest available region. This is further corroborated by studies that salmon did appear to prefer the warmest available region of a cage (up to 16°C) while avoiding the high light intensities during the day (Oppedal et al., 2011; Johnsen et al., 2020). Though most studies on this topic explore more extreme temperatures than have been presented here, these are representative of temperatures that Atlantic salmon are exposed to in Scottish waters.

When temperatures warmed to spring conditions and the water mixed, there was no longer a warmer depth, and the fish distributed themselves primarily in the centre of the cage. This is likely due to the trade-offs that animals contend with, among environmental and internal motivators such as perceived threats (Hoare et al., 2004; Oppedal et al., 2011). If the salmon perceive threats from above and below the cage, their natural inclination is to seek safety within the central area of the cage, where they can shoal with their cohort. This behavioural pattern implies that given temperature stratification within the cage, salmon tend to prioritise temperature preference, over safety from perceived threats. However, while the majority of fish were located in the deeper regions in Farms A and B, there were smaller sub-groups of fish occupying space in the centre region of the cage, seemingly preferring safety over temperature. These fish may be “reactive,” or shyer, choosing lower predation risk compared to the “proactive” fish that forego safety to swim in warmer waters (Castanheira et al., 2017). Fish which show reactive traits tend towards regions of safety within the cage while those with proactive traits may actively choose regions more exposed but have other benefits such as access to food or preferred temperatures. Moreover, laboratory studies have indicated that fish with different coping styles also show differences in temperature preferences, with proactive fish preferring higher temperatures compared to reactive fish, likely due to differences in basal metabolic rates (Cerqueira et al., 2016). An additional possibility is suggested by results from Johansson et al. (2006), that while salmon may all prefer the deeper waters, space constrains them, and a subsect of the population must inhabit other regions of the cage. If not, fish congregating in the warmer bottom waters where the net funnels can cause crowding and may have adverse effects for the fish. Basrur et al. (2010) examined the effects of weekly crowding on salmon and found that this stressor increased plasma cortisol and negatively affected growth rate and feed conversion ratio, though the magnitude of this impact was reduced over time, either implying habituation or a chronic stress effect. Alternatively, it is possible the fish shift from one area to the other based on different environmental and social interactions. However, as this study did not involve tagging the fish individually it is unknown whether it is the same fish choosing the bottom or the centre, thus further research on site fidelity should be done to clarify this point.

Photoperiod is an important behavioural driver to consider as well, as it is related to temperature. These two factors often exhibit a natural correlation, especially in temperate regions, where longer daylight hours during spring and summer coincide with rising water temperatures. As air temperatures increased to spring conditions, the thermocline dissipated in Farm A and the fish moved towards the centre and upper regions of the cage, towards the warmer UWC. However, the fish in all the farms began to ascend around the same time (early March), consistent with previous studies that suggest this may be linked with increased appetite in the spring due to the longer daylight hours, possibly influenced by changes in the circadian rhythm (Fernӧ et al., 1995; Oppedal et al., 2001; Oppedal et al., 2011). When examining the photoperiod, while Farm A had warmer UWC temperatures towards the end of the study period, the fish ascended with the length of the photoperiod. Farm B, however, had a higher range in photoperiod throughout the study, however the fish remained deeper compared to Farm A, indicating that temperature may have more of an effect on fish distribution compared to length of daylight hours. Fish in Farm C, which had no temperature gradients, used more of the water column when daylight hours increased, moving both up and down in the water column rather than shoaling towards the centre of the cage. This may be due to the general increase in metabolism and swimming activity that occurs when daylight hours expand (Al-Emran et al., 2024).

### 4.2 Horizontal distribution

At Farms B and C in January–May 2024, fish periodically showed significant higher activity levels on one side of the cage compared to the other indicating a preference for this location. The likely causes for this preference appear to relate to the wind and wave conditions at this time and differed between the farms that experienced the same storm event.

In both farms B and C, weather and current meters were deployed for the duration of the study. At the end of January 2024, Storm Isha passed over northwestern Scotland, characterised by a drop in atmospheric pressure to 953 hPa. This resulted in higher wind speeds and wave heights at the two farms on the Outer Hebrides. Subsequently, there were differing behaviours likely dependent on the site location and corresponding cage depth. It has been shown that salmon prefer to use the entire water column, only choosing specific regions as a response to temperature, currents, cage deformation, waves and daylight (Johannesen et al., 2022). At Farm B, the fish were located at the bottom of the cage, with warmer temperatures, avoiding the high wave heights. There is evidence that fish can experience motion sickness when in storm events, thus likely avoid regions of high wave heights (van de Vis et al., 2020). However, in Farm C, where the fish were in a shallower cage, they relocated to the area that was likely more sheltered due to the proximity to the other cages at the farm. There may have been slight cage deformation on the more exposed side of the cage as well. However, current speeds at these sites were relatively low (<0.2 m s^−1^), which has been shown to have only moderate changes in net deformation (5%; Lader and Enerhaug, 2005). This study corroborates a previous study by Johannesen et al. (2022) that demonstrated a 50-percentage point increase in the median proportion of time salmon were observed in the sheltered regions when wave heights were elevated, compared to the exposed side. Overall, this may constrain the usable space within the cage, potentially leading to overcrowding and negative impacts on oxygen levels. Moreover, during the storm event, a decline in SFR was observed, suggesting higher stress levels compared to Farm B, where no such change in SFR was detected. At these sites, feeding was remotely controlled from land, with feed operators monitoring fish behaviour and excess falling feed pellets via video feeds to determine when to stop feeding. This difference in feeding response may indicate that salmon in shallower cages, which offer less vertical space to avoid higher wave heights, are more prone to stress. However, more research is needed into this change in feeding response due to storms and whether this may be alleviated with deeper cages where fish can avoid these higher wave heights and associated stress.

### 4.3 Study limitations and considerations

This study was conducted using non-infrared cameras, thus further research is needed to investigate how environmental factors influence fish distribution during nighttime. Additionally, a limitation is the size of the cages relative to the number of cameras deployed. In large aquaculture cages, achieving full coverage is challenging, as complete observation with consistent illumination throughout the day is rarely feasible. Consequently, some behavioural data are inevitably missed. Alternative technologies, such as sonar, provide broader spatial coverage but lack the resolution of cameras. Combining both cameras and echosounders could offer a more comprehensive understanding and confirm the findings presented here. However, in practice, the current industry standard relies on a single camera per cage, using a subsample to infer population-level behaviour. By utilising four to five cameras, this study significantly extends the observed range, offering a more comprehensive assessment than is typically achieved in commercial settings. Lastly, a potential concern is that, given the upward-facing orientation of the cameras, fish from multiple depths could be visible in the footage, potentially influencing the interpretation of depth-specific behaviour. However, the cameras’ field of view is such that fish are only captured within the designated area of the water column where they are deployed (e.g., upper or lower water column). While some overlap in visibility may occur, the focus range of the cameras ensures that the observed activity is representative of the respective depth strata.

## 5 Conclusion

This study provides insight into the thermal preferences of Atlantic salmon in Scottish aquaculture, offering valuable information to farmers. Through extensive data collection utilising camera systems with an associated machine learning algorithm, a comprehensive baseline for fish distribution in relation to temperature was established, making it easier to identify a stress-related change, in this case due to a storm. In this study, winter temperature stratification resulted in heterogenous Atlantic salmon distributions, with fish preferring warmer temperatures in the LWC compared to the cooler UWC. As spring emerged, and photoperiod increased, the fish ascended and distributed themselves evenly throughout the cage. Unlike previous studies which have large temperature fluctuations, the findings of the study highlight salmon temperature preference under typical Scottish conditions, with minor temperature variations, depicting fish ability to discern slight changes in water temperature. These preferences can guide farmers in understanding salmon behaviour across different environmental conditions, offering critical insights for farmers who cannot continuously monitor all fish, ultimately improving fish welfare. Recognising depth preferences also serves as an indicator of stress, as deviations from normal patterns may signal distress. This was highlighted during a storm event, which appeared to influence the horizontal distribution of the fish. Fish response differed between the site with deeper cages compared to the shallower site. In the deeper cages, the fish were able to escape higher wave heights due to their position in the warmer waters in the LWC, while in the shallower cages, the fish moved towards the more sheltered side of the cage, closer in proximity to the other cages at the site. To enhance fish welfare, future research could consider designing cages that can be moved both vertically and horizontally to align with fish preferred temperatures and oceanographic conditions, while also considering management strategies that account for the behavioural differences between reactive and proactive fish.

## Data Availability

The datasets presented in this study can be found in online repositories. The names of the repository/repositories and accession number(s) can be found below: DataSTORRE http://hdl.handle.net/11667/242.

## Appendix

**TABLE A1:** Mean and median errors between the AI algorithm and human labellers.

Metric	Activity (%)
Mean error	−2.6
Mean average error	9.81
Median error	7
